# ML323 suppresses the progression of ovarian cancer *via* regulating USP1-mediated cell cycle

**DOI:** 10.3389/fgene.2022.917481

**Published:** 2022-07-18

**Authors:** Baozhi Song, Yatao Jiang, Yu Jiang, Ying Lin, Jiahua Liu

**Affiliations:** ^1^ Shengli Clinical Medical College of Fujian Medical University, Fuzhou, China; ^2^ Department of Gynecology, Fujian Provincial Hospital, Fuzhou, China; ^3^ Department of Obstetrics, Fujian Provincial Hospital, Fuzhou, China; ^4^ Department of Pathology, Fujian Provincial Hospital, Fuzhou, China

**Keywords:** ovarian cancer, ML323, deubiquitinase, USP1, cell cycle

## Abstract

**Background:** Ubiquitin specific protease 1 (USP1) tightly correlates with poor prognosis of multiple cancers. However, whether USP1 underlies ovarian cancer (OV) progression remains unclarified.

**Methods:** First, GSEA strategy and WGCNA analysis were used to screen for anti-ovarian cancer drugs and furthern optimal module, respectively. In addition, functional enrichments of module genes were realized by Gene Ontology (GO) and Kyoto Encyclopedia of Genes and Genomes (KEGG) pathway enrichment analysis. Kaplan-Meier was then employed to analyze the prognostic impact of USP1 expression on OV patients. Cell proliferation and cell cycle assays were used to confirm biological functions of USP1 in the final.

**Results:** Through the forementioned methods, we obtained five candidate drugs against OV from 353 anticancer drugs, and proposed ML323 as a novel anti-OV drug. As our hypothesized, ML323 significantly inhibited the proliferation of OV cells. Combined with WGCNA and KEGG analysis, the turquoise module was related to ML323, together with cell cycle. USP1 was subsequently identified as a target of ML323 and according to the TCGA database, USP1 negatively correlated with prognosis in OV, and its reduction and ML323-treatment both inhibited the proliferation of OV cells, blocking the S phase of cell cycle *in vitro*.

**Conclusion:** Taken together, ML323 exerts its inhibitory effect on the proliferation of OV cells by targeting USP1-regulated cell cycle, providing a therapeutical strategy and potential target against OV.

## Introduction

Ovarian cancer (OV) is the most lethal gynecological carcinoma, occupying the second mortality among women globally (Siegel et al., 2019). Despite considerable advancements in current surgical and chemotherapy treatment, the prognosis for ovarian cancer patients remains unfavorable, with a 5-years overall survival rate of less than 45% (Momenimovahed et al., 2019). (Simone et al., 2016), duing to its rapid tumor progression, metastasis, and limited therapeutic strategies (Torre et al., 2018). (Rottmann et al., 2017). Therefore, a deeper understanding of OV pathophysiological process and its underlying mechanism is the need-to-be-satisfied need.

Current researches continuously strive to reveal the “mysterious mast” blocking OV clinical therapy, including its molecular mechanisms, cancer progression, innate and acquired resistance to traditional treatments (Naumann et al., 2019). In 2014, bevacizumab, a vascular endothelial growth factor (VEGF) inhibitor, was approved to treat ovarian cancer for the first time (Pujade-Lauraine et al., 2014), and benefit from targeting angiogenesis, suppressing ovarian (and other) cancers from impeding their growth and spreads. Polyadenosine diphosphate ribose polymerase (PARP) inhibitor has been proved to be effective against ovarian cancer by DNA damage repairment. Olaparib was claimed to be the first FDA-approved drug (Kaufman et al., 2015). Various targeted drugs have been currently applied in OV treatment (Konstantinopoulos et al., 2019) (Moore and Monk, 2016), however, the mortality among OV patients continuesly raised annually (Webb and Jordan, 2017), Thus, an urgent need to discover effective targeted drugs for ovarian treatment remains in requirment to achieve better treatment outcomes.

Gene set enrichment analysis (GSEA), as a widely employed statistical techniques (Subramanian et al., 2005) (Subramanian et al., 2005), integrates physiological status and then creates *a priori* gene set to pair consistent behaviour under different biological conditions with different phenotypes or genotypes. Therefore, it is possible to evaluate co-regulatory genes with their function-related conditions, or other hypothetical biological linkage (Croken et al., 2014) statistically.

Ubiquitination is a crucial form of protein post-translational modification (PTM) that regulates the quantity and quality of diverse proteins and its substrate degradation, providing assurance of cellular homeostasis and life activities (Deng et al., 2020). In opposite, deubiquitinases (DUBs) catalyse the removal of ubiquitin (Das et al., 2020). Protein ubiquitination and deubiquitination participates multiple essential signalling networks and cellular pathways, including these relevant to cancers. Therefore, the success of bortezomib, a protease inhibitor, in the treatment of multiple myeloma in the clinic (Chen et al., 2011) stimulates interest in ubiquitination of proteins, suggesting eubiquitination as a potential anti-cancer therapeutic strategy (Scholz et al., 2020) (Mattern et al., 2012).

In this study, we first screened the differentially expressed gene set of ovarian cancer, and the target gene set of anti-tumour drugs by GSEA analysis to obtain anti-tumour drugs against ovarian cancer. We hypothesized ML323, a small molecule compound that can be used for ovarian cancer treatment and further explored the underlying mechanism of ovarian cancer. Subsequently, USP1 was identified as a direct downstream target gene of ML323. Finally we analyzed the target genes and related pathways of targeted drugs by WGCNA analysis and pathway enrichment, and validated their mechanism of action by relevant *in vitro* experiments.

## Materials and methods

### Data collection and samples collection

A total of 279 genome-wide expression matrices of ovarian cancer patients were downloaded from TCGA-OV (https://portal.gdc.cancer.gov/), and 88 genome-wide expression matrices of normal ovarian epithelial samples were downloaded from GTEx (https://xenabrowser.net/hub/). The target genes of 353 antitumor drugs/small molecules were obtained from the SEA database (https://sea.bkslab.org/). Data of ovarian cancer cell lines dependent on USP1 gene expression were obtained from the Depmap database (https://depmap.org/portal/). ML323 responses on ovarian cancer cells data from GDSC database (www.cancerRxgene.org).

Normal ovary tissue samples and ovary cancer tissue samples were taken at the Fujian provincial hospital from February 2020 to November 2020. Tissue sample collection was approved by the Internal Review and Ethics Boards of Fujian Provincial Hospital.

### WGCNA analysis and pathway enrichment analysis

In gene networks that follow a scale-free distribution, genes with comparable expression patterns could be co-regulated, functionally related, or share a pathway. WGCNA (version 1.7.1) method was adopted to construct the networks for the diseased groups and the control groups respectively. Briefly, for gene pairs, Pearson’s correlation coefficients (PCCs) or Spearman’s correlation coefficients (SCCs) were computed. Then, a weighted network adjacency matrix was calculated by increasing the absolute values of the correlation matrix to the power of β, resulting in a scale-free network. Genes in the above groupings were then grouped into selected modules by calculating the corresponding topological overlap.

### Cell lines culture

Human ovarian cancer cell lines OVCAR8, EFO21 were purchased from ATCC, and human normal ovarian epithelial cells IOSE-80 was obtained from Shanghai Yaji Biotechnology Co., Ltd. Based on the ATCC protocols, all cell lines were grown in the indicated medium and supplemented with 1% antibiotics (100 mg/ml streptomycin and 100 units/ml penicillin) and 10% (v/v) fetal bovine serum (FBS) at 37°C in a humidified incubator with 5% CO_2_.

### siRNA transfection

In 60 mm dishes, cells were plated at 60–70 percent confluency. OVCAR8 and EFO21 were transfected with si-USP1 or with a nontargeted siRNA as a control. GenePharma (Shanghai, China) provided the siRNA oligonucleotides, and the following are the sequences of the siRNA used in this study: si-USP1#1: GUAUACUUCAGGUAUUAUAdTdT; UAU​AAU​ACC​UGA​AGU​AUA​C dTdT; si-USP1#2: CCAUACAAACAUUGGUAAAdTdT; UUUACCAAUGUUUGU AUGGdTdT. And negative control siRNA (si-NC) sequences were: UUC​UCC​GAA​CGU​GUC​ACG​UTT; ACG​UGA​CAC​GUU​CGG​AGA​ATT. The transfection processes were carried out using Lipofectamine^®^ RNAiMAX (Thermo Fisher Scientific, 13778030) based on the manufacturer’s protocols.

### Quantification real-time PCR

Trizol Reagent (Invitrogen, Carlsbad, CA, United States) was used to extract total RNA from OV cell lines, which was then reverse transcribed using the RT Reagent Kit gDNA Eraser (TaKaRa). Then the expression of cDNA was detected by SYBR-Green (TaKaRa) and qRT-PCR, with β-ACTIN acting as an internal reference. Primers are depicted below:

hUSP1 Forward (F): 5′-GCC​AAT​GAG​AGC​GGA​AGG​AG-3′;

hUSP1 Reverse(R): 5′-CAA​TTA​GAT​GGG​CGG​GAG​CA-3′.

hβ-ACTIN Forward (F): 5′-AGT​TGC​GTT​ACA​CCC​TTT​CTT​G-3′;

hβ-ACTIN Reverse(R): 5′-CAC​CTT​CAC​CGT​TCC​AGT​TTT-3′.

### Cell proliferation assay

OVCAR8 and EFO21 cells were plated in 96-well plates with a cell density of 2,000 per well and cultured for 2–3 days in 100 μl of complete culture medium. Cells were treated with an equivalent volume of DMSO as control. The cell counting kit-8 (CCK-8) assay was used to determine cell proliferation. At 0, 24, 48, and 72 h following drug treatment or transfection, cell proliferation was evaluated in OV cells using the cell counting kit-8 (CCK-8) assay (Dojindo Molecular Technologies, Inc.). Briefly, the OV cells were incubated for 1 h at 37°C with 10 µl CCK-8 reagent. Optical density values at 450 nm were used to calculate the cell proliferation values. This process above was repeated for 4 days. Each experiment was repeated three times.

### Cell cycle analysis

Before the cell cycle distribution test, OVCAR8 and EFO21 cells were synchronized to S phase using thymidine and treated with si-NC, si-USP1 and ML323 for 48 h. The Accuri C6 flow cytometer system was used to collect the cells. FlowJo software was used to analyze the data (Tree Star Inc., Ashland, OR).

### Statistical analysis

GraphPad 5.0, was used to conduct statistical analysis. The means ± S. E. M. or S. D. are used to represent the data. Student’s t test was applied to evaluate the statistical significance. Significant *p* values were defined as < 0.05.

## Results

### Small molecular drug ML323 protects against ovarian cancer

**FIGURE 1:**
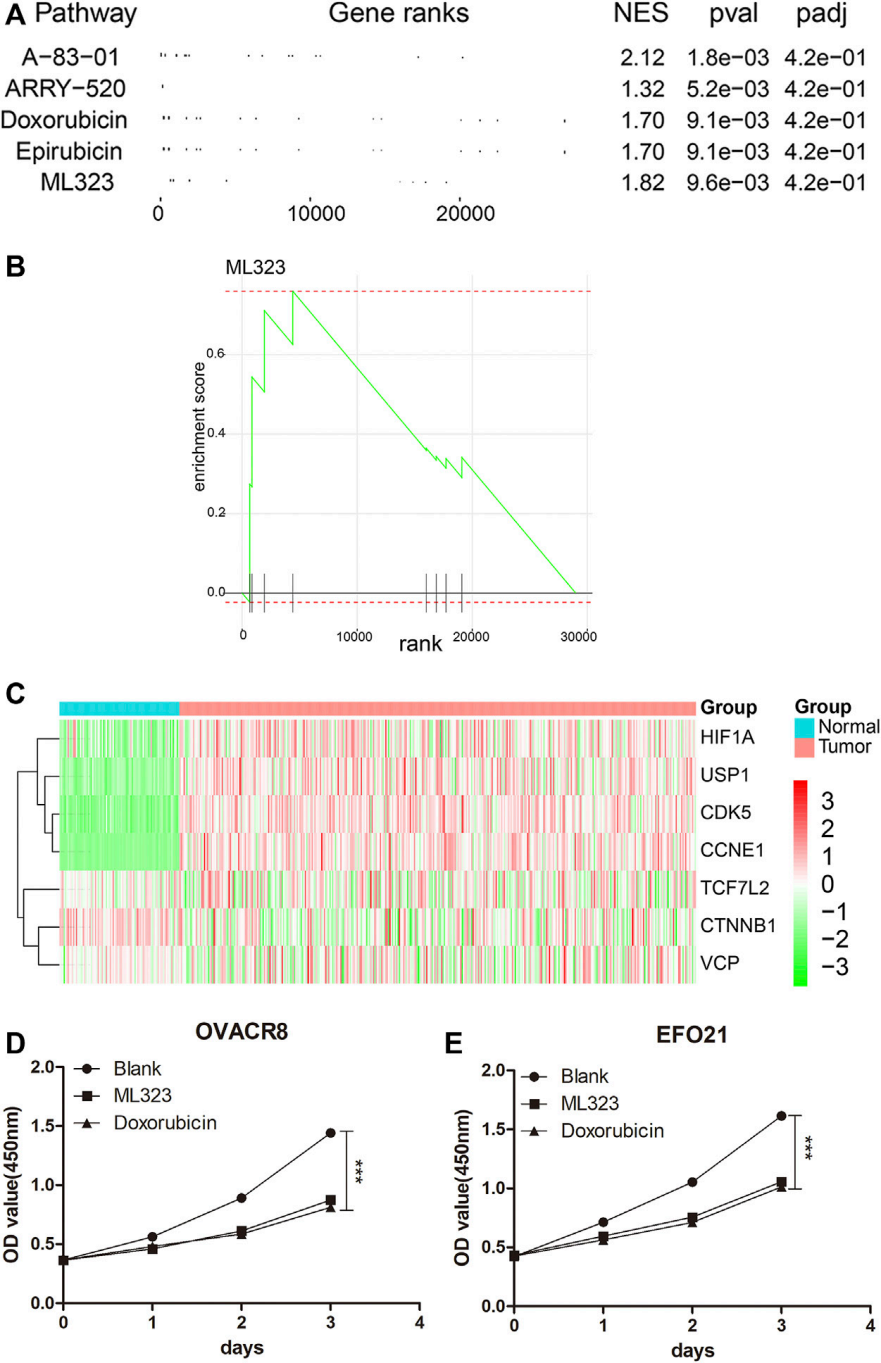
Screening for ML323, a small molecule drug for ovarian cancer. **(A)** The top five drugs with anti-ovarian cancer effects were obtained by GSEA analysis. **(B)** GSEA analysis of ML323 and gene sets in OV samples. **(C)** Heat map of the target genes of the small molecule drug ML323. **(D–E)** ML323 inhibited the proliferation of OVACR8 and EFO21 cells.

The flowchart diagram was constructed to display the methods and results of our study (Supplementary Figure S1). In order to screen for drugs with anti-ovarian cancer effects, we first extracted genome-wide expression matrices from 279 ovarian cancer patients in TCGA-OV (https://portal.gdc.cancer.gov/) and 88 normal ovarian epithelial tissues in GTEx (https://xenabrowser.net/hub/). The Bayesian t-values of the whole genome were obtained as rank by performing Bayesian tests with the “limma” package (version 3.52.0) of R software (version 4.0.2). Then, targeted genes of 353 anti-tumour drugs/small molecules were obtained from the SEA database (https://sea.bkslab.org/) as set files. The top five drugs with anti-ovarian cancer effects were obtained by GSEA analysis (Figure 1A). Among them, A-83–01 (Yamamura et al., 2012), ARRY-520 (Kim et al., 2009), Doxorubicin (Hainsworth et al., 1988) and Epirubicin (Cersosimo and Hong, 1986) were relevent to ovarian cancer. The above literature further supports the results of our analysis. Considering the novelty, we selected small molecular drug, ML323, as the further investigated object. As shown in Figure 1B, we obtained the enrichment map of ML323 by GSEA analysis. The SEA database (https://sea.bkslab.org/) shows target genes for anti-tumor drugs. We observed the target genes of ML323 as Figure 1C shown. Seven target genes in heat map have been listed in OV and normal control samples from the SEA database. In consistant with bioinformatic data, we observed *in vitro* experients that addition of 30 μM ML323 to OVACR8 and EFO21 cells inhibited cell proliferation (Figures 1D,E), and the inhibitory effect was approximated to the positive drug doxorubicin, suggesting the protective effect of ML323 on OV treatment.

### USP1 mediates the anti-ovarian cancer effect of ML323

**FIGURE 2:**
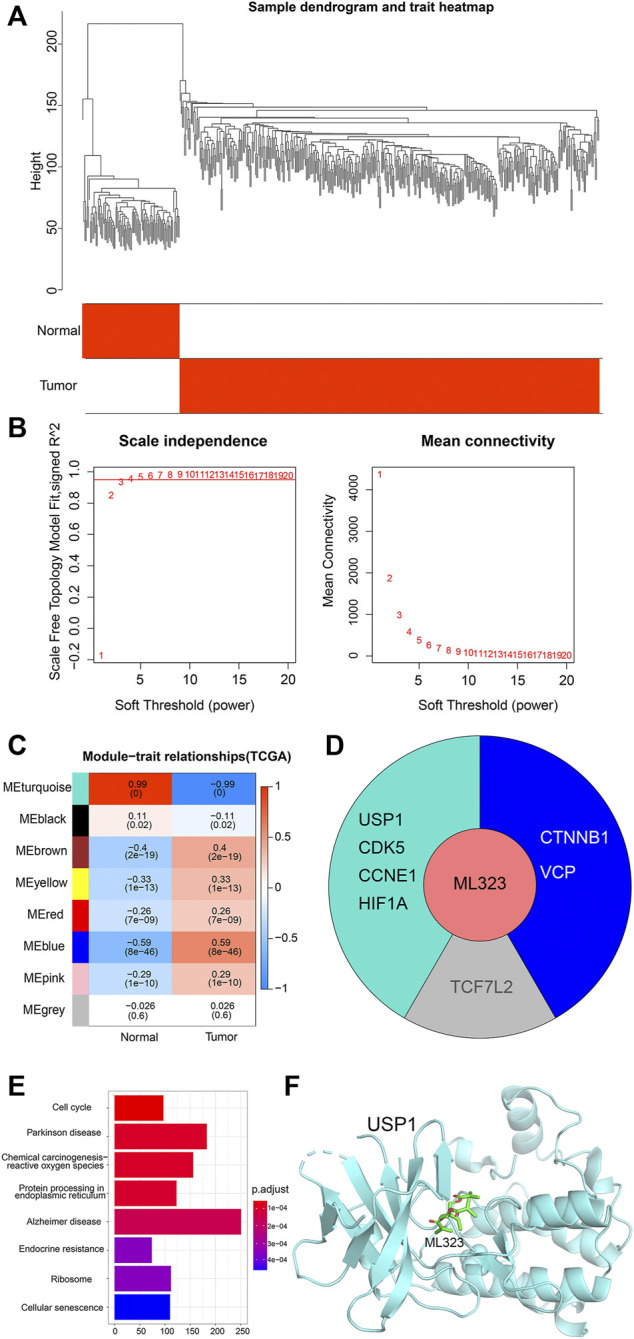
Construction of weighted co-expression network and identification of key modules. **(A)** Hierarchical clustering dendrogram of OV samples and normal ovary samples. **(B)** Analysis of scale-free fit metrics. **(C)** Correlation of modularity with normal group and OV group. **(D)** Modules distributed by the 8 target genes of ML323. **(E)** Pathway analysis of the turquoise module in which the four target genes of ML323 are located. **(F)** Molecular docking simulation of ML323 and USP1.

In order to understand the main modules of ML323 target gene enrichment, we performed WGCNA analysis on ovarian cancer and normal samples using the “WGCNA package” (Chen et al., 2020) of R software. As shown in Figure 2A, a dendrogram of the 279 OV samples in TCGA based on hierarchical clustering and 88 normal ovary samples in GTEx, with qualified classification of OV and normal ovary samples. Then, we defined the optimal soft threshold as 5 to meet the requirement of obtaining a scale-free network (Figure 2B). Subsequently, we analysed the relationship between modules and sample subgroups using Pearson correlation coefficients (r) and found that all gene modules were significantly correlated with OV and normal controls, except for the grey module (Figure 2C, *p* < 0.05). We also analyzed the distribution of the eight target genes of the small molecule ML323 in the modules and found that USP1, CDK5, CCNE1 and HIF1A were concentrated in the turquoise module (Figure 2D). USP1 was previously reported to be a potential target against NSCLC, whereas none relevant reports were found in OV. In this reason, we selected the turquoise module where USP1 was dominantly located in our current study for a further analysis. We performed KEGG pathway analysis on the turquoise module gene and found a significant association with cell cycle (Figure 2E). To achieve docking, we obtained the USP1 structural domain (PDB code: 4PWN) and the ML323 3D structural domain (PubChem CID: 18689). The AutodockTools-1.5.6 tool generated a series of docking patterns with energy scores ranging from 16.6623 to 34.6584, implying a favourable docking mode. Thus, a hypothetical docking pattern for ML323 and its target USP1 was confirmed (Figure 2F), sufficiently explained the reason we chose USP1 as the target gene for the treatment of OV.

### Overexpression of USP1 correlated with prognosis in OV patients

**FIGURE 3:**
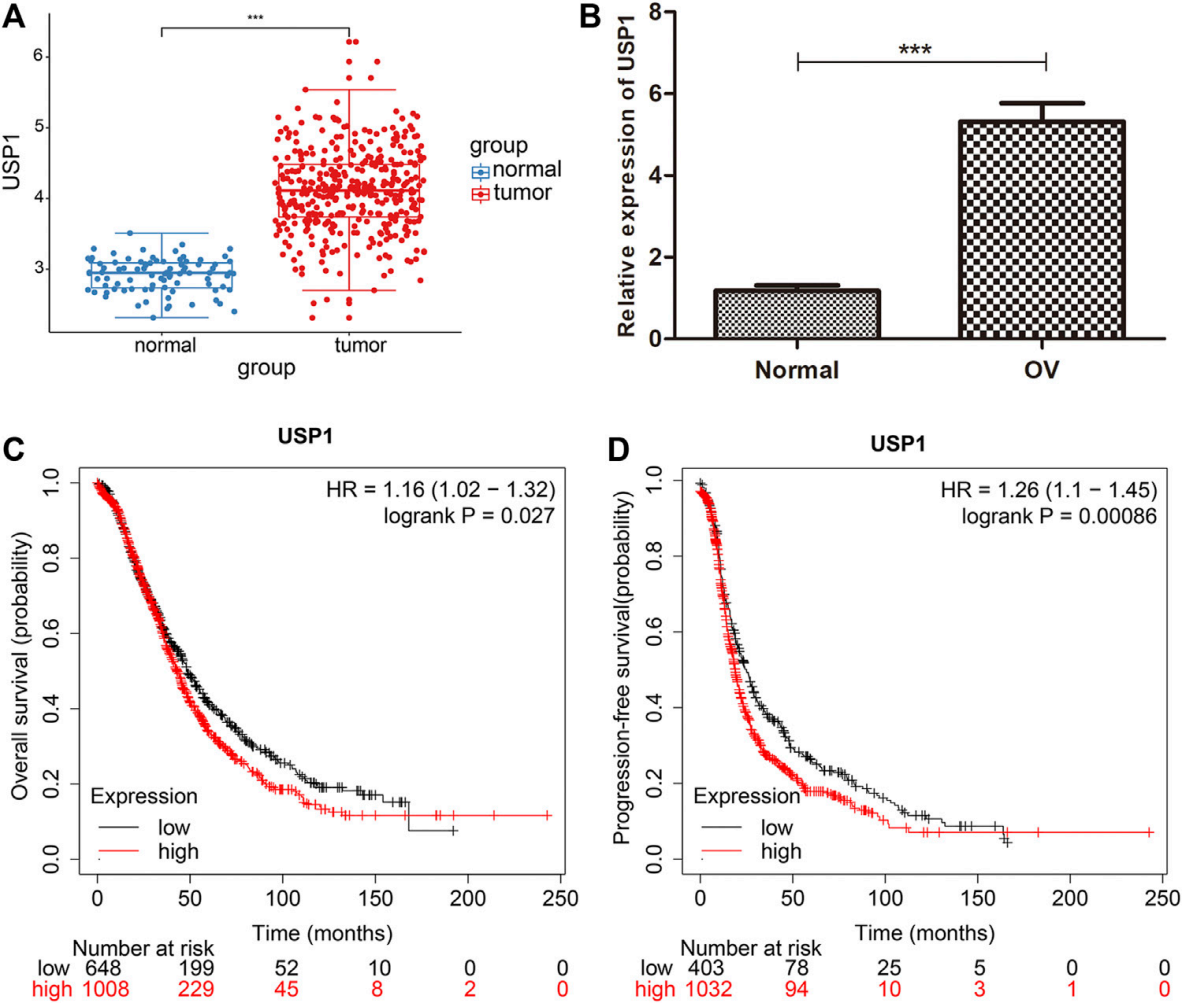
USP1 is highly expressed in OV and is associated with patient prognosis. **(A)** Expression of USP1 in OV tissues from TCGA database. **(B)** Real-time PCR analysis to quantify the levels of USP1 in 20 paired OV tissues. **(C)** Kaplan-Meier survival curves for overall survival of patients with high and low USP1 expression. **(D)** Kaplan-Meier plots of progression-free survival in patients with high and low USP1 expression.

In order to address the difference of USP1 between ovarian cancer and normal ovarian epithelial tissue, we analyzed ovarian cancer samples in TCGA and normal ovarian epithelial samples in GTEx, and plotted the box plots of expression (Figure 3A, *p* < 0.001). In order to comprehensively understand, we examined USP1 mRNA expression in 20 OV samples and normal ovarian tissues by qRT-PCR. Comparing to the normal, USP1 expression was considerably increased in OV samples (Figure 3B). The expression pattern of USP1 in OV patients was then examined by using Kaplan-Meier’s survival analysis, and demonstrated that upregulation of USP1 was substantially linked with poor patient prognosis (Figures 3C,D). In total, these findings revealed that USP1 overexpression might be applied to predict OV prognosis.

### Knockdown of USP1 inhibited OV cellular proliferation

**FIGURE 4:**
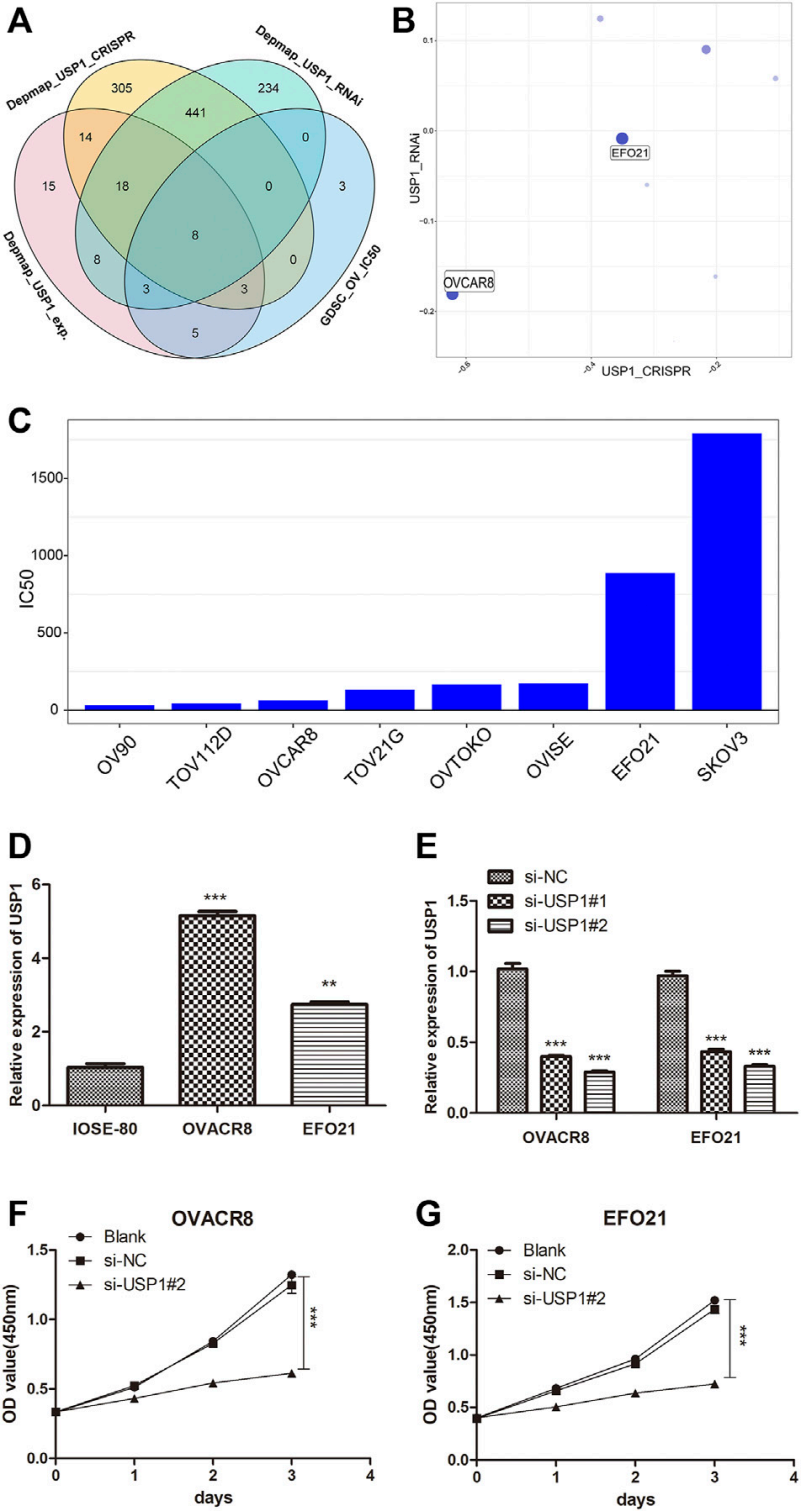
USP1 silencing inhibited OV cell proliferation. **(A)** Venn diagram of screening ovarian cancer cell lines. **(B)** Bubble diagram of combined CRISPR and RNAi evaluation of ovarian cancer cell lines. **(C)** Histogram of IC50 values of ovarian cancer cell lines against the small molecule ML323. **(D)** USP1 expression levels in the normal ovarian cell line and ovarian cancer cell lines were detected by quantitative PCR. **(E)** Validation of OVACR8 and EFO21 cells with USP1 knockdown by qPCR. **(F–G)** Proliferation rate of OVACR8 and EFO21 cells detected by CCK-8 analysis.

In order to further explore the regulatory mechanism of ML323/USP1 in ovarian cancer, we downloaded 74 ovarian cancer cell lines with averaged USP1 expression, 789 ovarian cancer cell lines with CRISPR-treated dependency index of the USP1 gene, and 712 ovarian cancer cell lines with RNAi-treated dependency index of the USP1 gene from the Depmap database (https://depmap.org/portal/). At the same time, 22 ovarian cancer cell lines with IC50 values after ML323 treatment were obtained from GDSC database. The above data were intersected and we obtained 8 cell lines (Figure 4A), and we finally selected OVACR8 and EFO21 for subsequent experiments by the mean expression of USP1, CRISPR and RNAi combined evaluation (Figure 4B). The IC50 of aforementioned eight cell lines were also depicted, and OVACR8 and EFO21 were shown to be more susceptible to ML323 (Figure 4C). In order to perform functional analysis, the expression of USP1 was determined in ovary cancer cell lines. Comparing to normal ovary epithelial cells, the expression of USP1 was three to four-fold higher in the OV cell lines (Figure 4D). A set of siRNA stably transfected with OVACR8 and EFO21 cells were used to knockdown the expression of endogenous USP1, as shown in Figure 4E siUSP1-#2 effectively inhibited the expression of USP1 as for the reason to be chosed in subsequent tranfection. We knocked down USP1 in OV cells and assessed cell proliferation by using the CCK-8 assay. Comparing with si-NC cells, knockdown of USP1 in the OVACR8 and EFO21 cells showed decreased cell proliferation (Figures 4F,G). Taken together, our study found that downregulated USP1 decreased OV cell proliferation.

### Downregulation of USP1 blocked OV cell cycle *in vitro*

**FIGURE 5:**
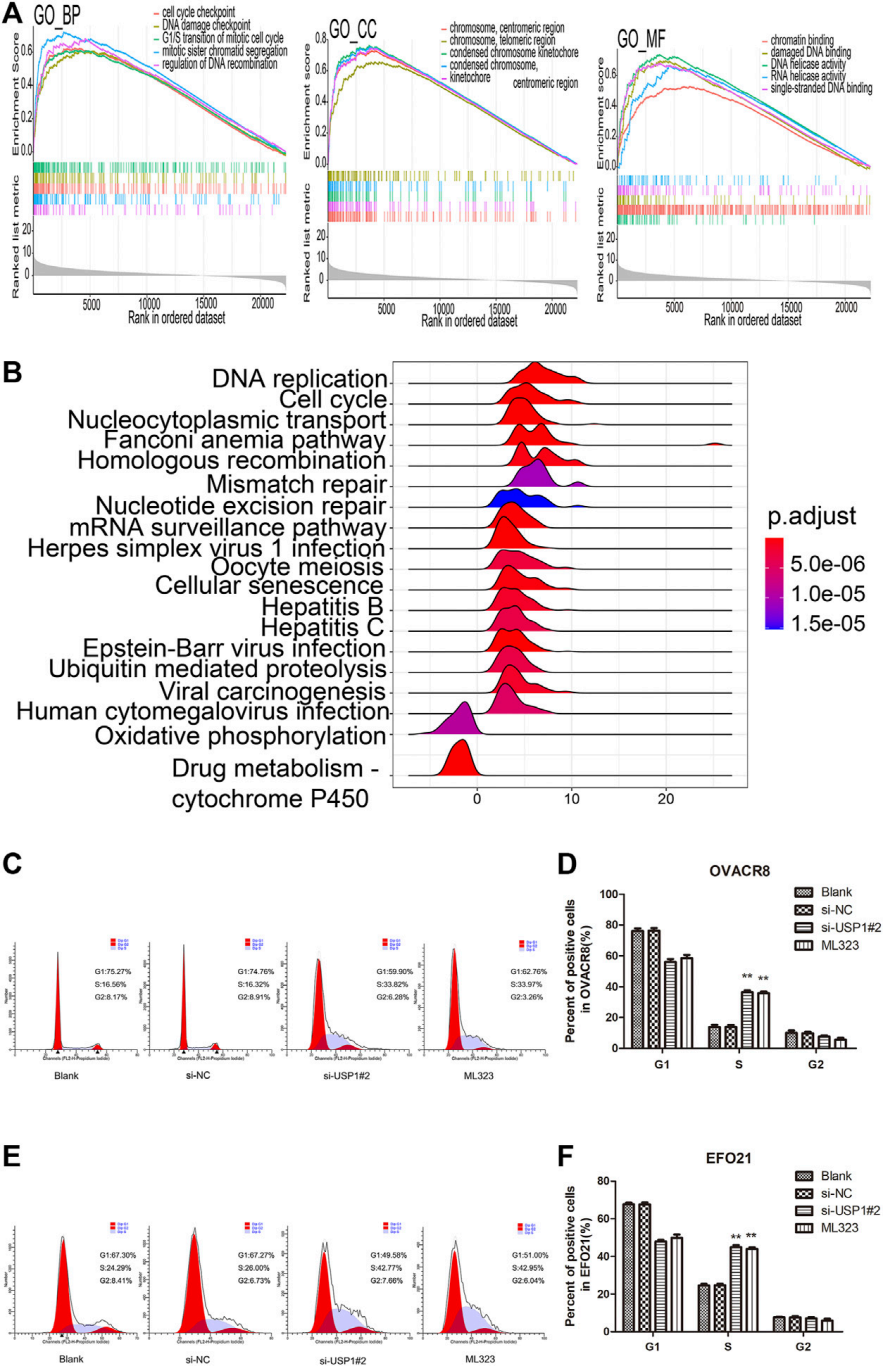
USP1 silencing blocked the OV cell cycle. **(A)** GSEA of CC, BP, MF of GO pathway analysis, respectively. **(B)** KEGG pathway enrichment analysis of genes co-expressed with USP1. **(C–D)** Cell cycle assays were analysed for representative images (left) and quantification (right) of blank, si-NC, si-USP1-transfected and ML323-treatment OVACR8 cells. **(E–F)** A representative image (left) and quantification (right) of blank, si-NC, si-USP1-transfected and ML323-treatment EFO21 cells were analysed in cell cycle experiments.

To gain the insight of the underlied molecular process of ML323/USP1 axis, we divided the 279 patients of TCGA-OV into up-regulated and down-regulated groups according to the median expression of USP1, and performed Bayesian analysis by using the R software “limma” package to distinguish significantly different genes (|log2FC|>1, *p* < 0.05, FDR<0.05) for GO/KEGG pathway analysis. Figure 5A shows the GSEA of CC, BP, MF of GO pathway analysis, respectively, and Figure 5B exhibits the KEGG pathway analysis, indicating a strong correlation of USP1 with cell cycle. Knocking down of USP1 in OVACR8 and EFO21 cells was performed to confirm the results of the aforementioned bioinformatics investigation. We also examined the cell cycle of OVACR8 and EFO21 cells in context of ML323. Cell cycle was then analyzed by using flow cytometry (Figures 5C–F). We observed that USP1-downregulated OVACR8 and EFO21 OV cell cycles were arrested in S-phase. The brief summary was that in comparison to blank control and si-NC cells, OV cells with USP1 knockdown and ML323-treatment showed higher extent arrest in S phase. These observations suggested that USP1 was required for S-phase cell cycle progression and proliferation in OV cells. Further, ML323 targets USP1, thereby inhibiting the proliferation of OV cells by blocking the progression of the OV cell cycle, thus achieving inhibition of tumor proliferation.

## Discussion

In the present research, we proposed small molecule compound ML323 as a target drug against ovarian cancer for the first time by GSEA analysis. ML323 was found to dramatically inhibit cell proliferation in ovarian cancer cell lines. Additionally USP1, a deubiquitinase, was found to be upregulated comparing OV tissues to normal tissues,. and negative related to poor patient prognosis. Functional studies showed that inhibition of USP1 blocked OV cell proliferation and cell cycle. Furthermore, we identified USP1 as a key downstream target gene of ML323, which is responsible for inhibiting OV cell proliferation and affecting OV cell progression through inhibition of USP1.

ML323 is a small molecule inhibitor of USP1-UAF1 and targets two major DNA damage response pathways simultaneously by inhibiting USP1 (Liang et al., 2014), suggesting its potential against various malignancies in previous researches. USP1 was shown to be upregulated in ovary cancer cells and tissue samples. The overexpression of USP1 may hint high oncogenic risk in ovarian carcinogenesis. In multiple cancers, USP1 has been identified as a key oncogene. Upregulation of USP1 is related to progression and poor prognosis such as, multiple myeloma (Das et al., 2017), and hepatocellular carcinoma (Zhao et al., 2020), and also mediated chemotherapy resistance (Sonego et al., 2019) (García-Santisteban et al., 2013). Overexpression of USP1 increases the expression of several prometastatic genes in breast cancer cells, promote cell migration and invasion, thereby promoting metastasis of breast cancer cells (Ma et al., 2019). In our current study, we have also addressed that ML323 inhibits the proliferation of ovarian cancer cells and ovarian cancer progression. Similarly, knockdown of USP1 also inhibited the proliferation of ovarian cancer cells. In addition, studies have shown that USP1 was found in the nucleus and regulated both cell cycle progression (Cotto Rios et al., 2011) and DNA damage response. Cell cycle regulates the transcription of the USP1 gene in a cell cycle-dependent manner, with USP1 mRNA remaining relative low in the G1 phase and peaking in the S phase (García-Santisteban et al., 2013). In the current study, we discovered that knocking down USP1 blocked the cycle of ovarian cancer cell lines in the S phase, consistent with above mentioned findings. USP1 is a regulator of multiple key processes during DNA damage response, most notably in the Fanconi depletion (FA) pathway (Nijman et al., 2005) and translesion synthesis (TLS) (Huang et al., 2006). USP1 deubiquitinates ub-FANCD2 (Nijman et al., 2005) and ubFANCI (Sims et al., 2007), thereby restoring FA key events in pathway activation. The key substrate for USP1 in TLS belongs to proliferating cell nuclear antigen (PCNA) (Nijman et al., 2005). USP1 deubiquitylates mono-ubiquitylated PCNA, which inhibits DNA polymerases recruiting in the absence of DNA damage, resulting in regulating DNA repair. Furthermore, inhibition of USP1 increased the sensitivity of colorectal cancer cells to DNA-damaging chemotherapeutic agents (Xu et al., 2019). Therefore, inhibition of USP1 expression possibly improve prognosis and increase the sensitivity of chemotherapeutic agents, providing a potential strategy for oncology treatment.

There are several limitations to our study that need to be explained. Firstly, all the data of patients used in this study obtained from the same TCGA cohort and the sample size was relative small. For further accurate conclusion, a larger sample cohort was in need. Secondly, the molecular mechanism exploration of this paper mainly based on bioinformatics approaches such as enrichment analysis. The experimental part was relative limitied, only the *in vitro* validation. *In vivo* experiment inclusion may provide more sufficient proof.

In summary, our work has identified the critical role of ML323/USP1 axis against anti-ovarian cancer. According to the findings, ML323 could be a reliable anti-ovarian cancer agent though targeting USP1. Further, USP1 could be a prognostic marker and anti-cancer therapeutic target for ovarian cancer.

## Data Availability

The datasets presented in this study can be found in online repositories. The names of the repository/repositories and accession number(s) can be found in the article/Supplementary Material.
