# Supplementary figures and images for: ML323 suppresses the progression of ovarian cancer via regulating USP1-mediated cell cycle
Source: Front Genet. 2022 Jul 18;13:917481. doi: 10.3389/fgene.2022.917481 (PMC9340375; doi:10.3389/fgene.2022.917481)

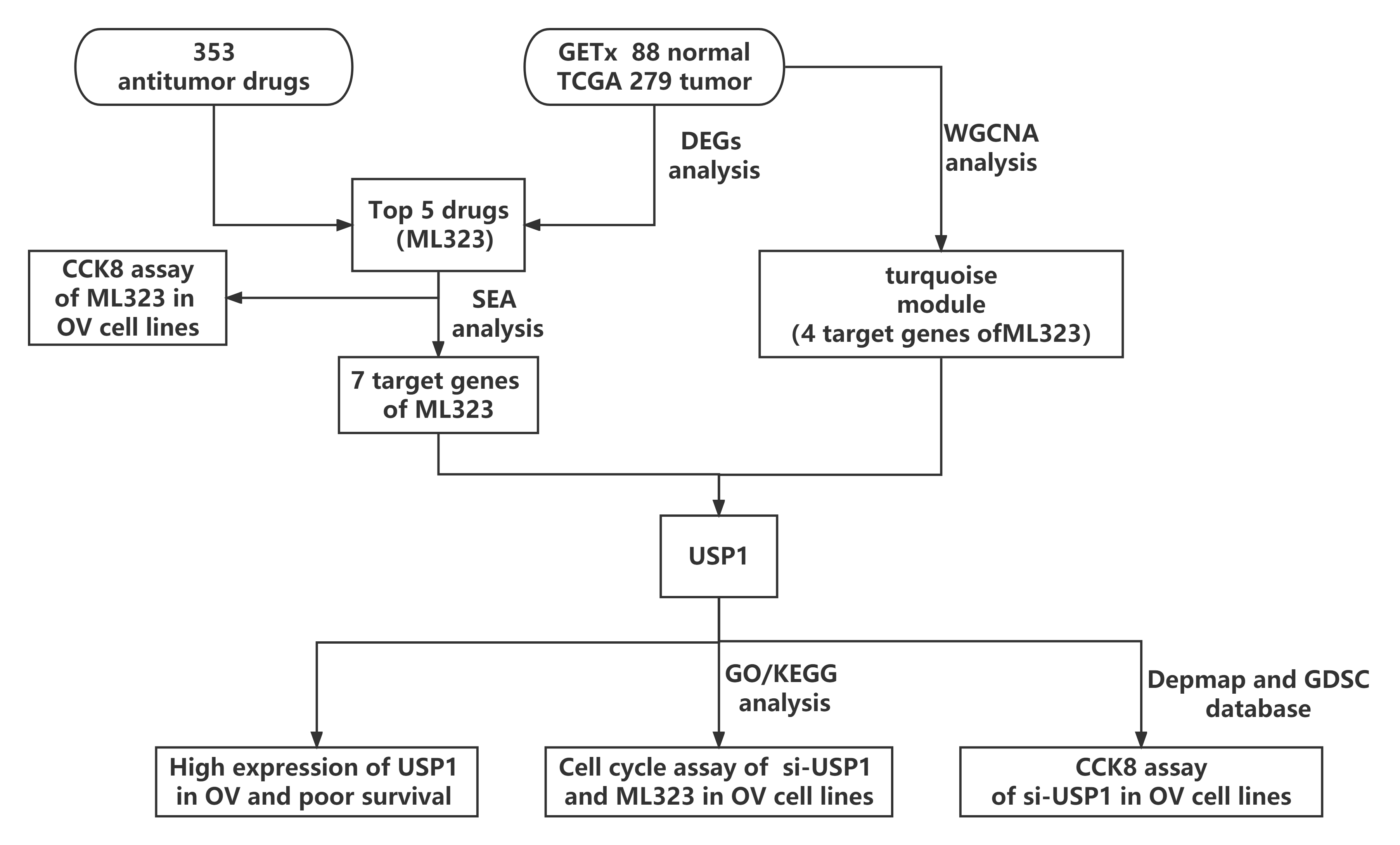

Supplement: Supplementary file 1 [file Image1.TIF]
